# Novel Melanocortin 2 Receptor Variant in a Chinese Infant With Familial Glucocorticoid Deficiency Type 1, Case Report and Review of Literature

**DOI:** 10.3389/fendo.2019.00359

**Published:** 2019-06-06

**Authors:** Kuerbanjiang Abuduxikuer, Zhong-Die Li, Xin-Bao Xie, Yu-Chuan Li, Jing Zhao, Jian-She Wang

**Affiliations:** Department of Hepatology, Children's Hospital of Fudan University, Shanghai, China

**Keywords:** melanocortin 2 receptor (*MC2R*), familial glucocorticoid deficiency (FGD) type 1, cholestasis, skin hyperpigmentation, hypoglycemia, cortisol, adrenocorticotropic hormone (ACTH), linear overgrowth

## Abstract

Familial glucocorticoid deficiency type 1 (FGD1) is an autosomal recessive disorder caused by mutations in the melanocortin 2 receptor (*MC2R*) gene, characterized by a low or undetectable serum cortisol level and a high adrenocorticotropic hormone (ACTH) level. Clinical manifestations include hypoglycemia, seizure, skin hyperpigmentation, hyperbilirubinemia, cholestasis, and a tall stature. Some dysmorphic features such as, a prominent forehead, hypertelorism, a broad nasal bridge, and small tapering fingers, have been reported. Children with FGD1 may have other isolated endocrine abnormalities. To date, no patient with FGD1 has been reported in mainland China. Here we report on a Chinese patient with FGD1 having a novel MC2R gene variant, a mild transverse palm crease, hypertelorism, and subtle/transient endocrine abnormalities relating to all three zones of the adrenal cortex and thyroid gland. We also reviewed cases with dysmorphic features or additional endocrine abnormalities.

## Introduction

Familial glucocorticoid deficiency type 1 (FGD1) (OMIM #202200) is an autosomal recessive disorder caused by mutations in the melanocortin 2 receptor (*MC2R*) gene (OMIM ^*^607397), characterized by a low or undetectable serum cortisol level and high adrenocorticotropic hormone (ACTH) level. Clinical manifestations include hypoglycemia, seizure, skin hyperpigmentation, hyperbilirubinemia, cholestasis, and a tall stature (1–6). Other dysmorphic features, such as a prominent forehead with hypertelorism (7), and a broad nasal bridge with small tapering fingers (8), have been reported. Children with FGD1 may have other isolated endocrine abnormalities (4, 6–17). Here we report on the first FGD1 patient from China with a novel MC2R gene variant, a mild transverse palm crease, hypertelorism, and subtle/transient endocrine abnormalities relating to all three zones of the adrenal cortex and thyroid gland.

## Case Presentation

The female infant was born to healthy non-consanguineous parents (25-year-old father, and 22-year-old mother) after an uncomplicated first pregnancy and 40 weeks of gestation. A Cesarean section was performed due to a failed vaginal delivery, but the Apgar score until 15 min after birth and birth weight was normal (3,300 g). The patient was intubated after developing progressive tachypnea, moaning, and severe hypoglycemia (0.9 mmol/L). The patient developed hyperbilirubinemia that was unresponsive to phototherapy, but the parents took the baby home, for home care at the age of 9 days.

At the age of 1.4 months, the patient was admitted to a provincial hospital for jaundice, vomiting, afebrile seizures, and pneumonia. The lowest blood glucose level during the hospital stay was 1.4 mmol/L. The serum cortisol levels were extremely low (13.8–29.3 nmol/L, normal range 138–690 nmol/L) while adrenocorticotropic hormone levels were slightly lower or normal (6.0–18.5 pg/ml, normal range 6.4–40 pg/ml). A cortisol deficiency was diagnosed, but parents refused hormone replacement therapy. The patient was discharged after the pneumonia was resolved and blood glucose levels were stabilized.

**Figure 1:**
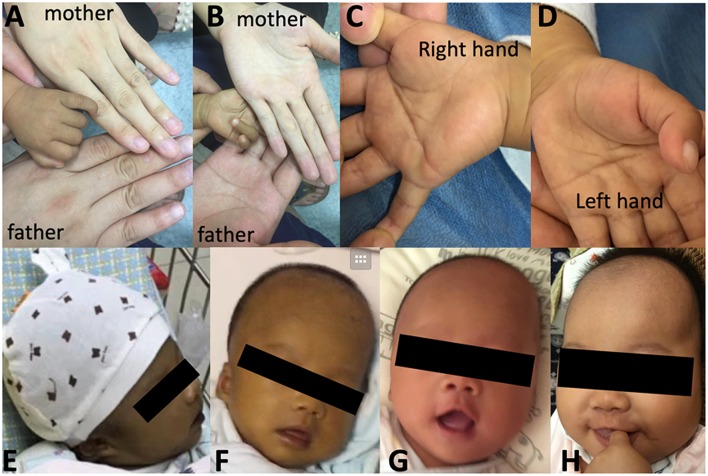
Skin pigmentation and palmar creases. Higher skin pigmentation of the hand compared to parents at 3.5 months **(A,B)**, a slight transverse palmar crease on the right hand **(C)** but not on the left hand **(D)**. Changes in skin pigmentation at 0.7 months **(E)**, 1.6 months **(F)**, 3 months **(F)**, and 4.9 months (**H**, 1.5 months after hydrocortisone therapy and resolution of cholestasis). Written informed consent was obtained from the parents for the publication of personal images.

At the age of 3.2 months, the patient was presented to our hospital for cholestasis without obvious symptoms of hypoglycemia, infection, alacrima, or achalasia. Repeated morning serum cortisol levels were extremely low (8.8–10.6 nmol/L, normal range 138–690 nmol/L), while ACTH was extremely elevated (1656.9–1911.8 pg/ml, normal range 6.4–40 pg/ml). Upon physical examination, significant jaundice, skin hyperpigmentation and slight hepatosplenomegaly (liver 2–2.5 cm below the right costal margin, and 2.5 cm below the xiphoid process; spleen 1.5–2.0 cm below the left costal margin) were observed. Slight dysmorphic features such as a transverse palmar crease in the right hand, a prominent forehead, hypertelorism (inner canthal distance greater than the palpebral fissure length) were noted. The palmar crease, and the changes in skin pigmentation are presented in Figure 1. Written informed consent was obtained from the parents for the publication of this case report and related images. Changes in body weight/length, complete blood count, procalcitonin, serum biochemistry, blood coagulation, and endocrine profiles throughout the disease course are provided in Table 1.

**Table 1:** Changes in body weight/length, complete blood count, procalcitonin, serum biochemistry, blood coagulation, and endocrine profiles.

**Age (d, day; m, month)**		**2 d**	**7 d**	**1.4 m**	**1.7 m**	**3.2 m**	**3.4 m^a^**	**3.8 m**	**4.2 m^b^**	**0.9 m^c^**	**8.1 m^d^**
Weight	Kg	3.3	–	4.0	–	6.3	6.6	–	–	9.0	13.0
	WHO percentile	38th	–	6th	–	39th	24th	–	–	96th	>99th
Length	Cm	51.0	–	–	–	–	65.5	–	–	70.0	77.0
	WHO percentile	63rd	–	–	–	–	69th	–	–	98th	>99th
Serum biochemistry (reference range)	Albumin (35–55 g/L)	28.9	31.2	34.0	33.4	37.9	36.1	41.9	42.3	44.9	39.0
	Globulin (20–30 g/L)	18.4	19.6	16.6	14.4	17.5	19.5	24.6	–	18.3	18.9
	Alanine aminotransferase (0–40 IU/L)	7	14	14	42	101	101	78	70	119	40
	Aspartate aminotransferase (0–40 IU/L)	50	34	56	167	320	359	155	66	83	61
	Total bilirubin (5.1–17.1 umol/L)	172.0	290.9	147.1	79.4	100.6	104.1	71.0	24.3	12.8	6.9
	Direct bilirubin (0–6umol/L)	13.6	16.2	37.3	46.5	63.1	68.6	47.3	15.5	7.0	1.8
	γ-Glutamyl transferase (7–50 IU/L)	377	306	274	192	54	49	68	59	67	12
	Total bile acid (0–10 umol/L)	–	–	–	–	441	475	323	–	–	18
	Alkaline phosphatase (42–383 IU/L)	146	171	373	376	742	812	540	342	283	–
	Blood glucose (3.9–5.8 mmol/L)	2.65	–	6.6	1.44	2.5	7.15	6.1	–	–	–
	Lactic acid (0–2 mmol/L)	–	–	–	3.0	–	3.6	–	–	–	–
	Ammonia (10–47 umol/L)	–	–	–	123	–	100	–	–	–	–
	Total cholesterol (3.1–5.2 mmol/L)	3.09	–	2.59	2.85	4.19	4.26	6.25	–	–	–
	Triglyceride (0.56–1.70 mmol/L)	0.8	–	–	–	–	1.72	2.46	–	–	–
	Creatine kinase (22–270 U/L)	1,259	–	292	–	–	279	–	–	–	–
	Creatine kinase-MB (2–28 U/L)	40	–	–	–	–	34.7	–	–	–	–
	Lactate dehydrogenase (100–240 U/L)	526	–	530	284	–	469	–	–	–	–
	Procalcitonin (<0.05 ng/ml)	–	–	–	–	17.4	–	7.7	13.4	–	–
Endocrine profiles (reference range)	Morning cortisol (138–690 nmol/L)	56.8	–	29.3	<13.8	8.8	10.6	–	29.5	242.5	1.35
	ACTH (8–10 Am, 6.4–40 pg/ml)	–	–	18.5	6.0	1656.9	1911.8	–	263.4	58.7	1999.9
	Renin (4–24 ng/ml/hour)	–	–	–	–	–	26.3	–	–	–	–
	Aldosterone (10–160 pg/ml)	253.6	–	113.8	–	–	192.7	–	–	–	–
	Angiotensin II (25–129 pg/ml)	–	–	–	–	–	149.5	–	–	–	–
	17-alpha hydroxyprogesterone (0.8–16.6 ng/ml)	–	–	0.4	–	–	0.4	–	–	0.009	–
	Androstenediol (0.3–3.3 ng/ml)	–	–	–	<0.3	–	0.3	–	–	<0.3	–
	Dehydroisoandrosterone (1–11.7 umol/L)	–	–	–	–	–	0.1	–	–	–	–
	Testosterone (0–1.08 nmol/L)	1.50	–	0.23	0.67	–	2.09	–	–	0.46	–
	Progesterone (0.1–0.33 ng/ml)	>60	–	0.18	0	–	–	–	–	0.04	–
	Dehydroepiandrosterone sulfate (35–430 ug/dl)	–	–	–	<15	–	–	–	–	–	–
	C peptide (0.3–3.73 ng/ml)	0.23	–	0.03	–	–	–	–	–	–	–
	Insulin (4.03–23.46 uIU/ml)	4.86	–	0.34	–	–	–	–	–	–	–
	Thyroid-stimulating hormone (0.25–7.31 mIU/L)	–	10.61	4.15	–	–	4.81	–	–	–	4.87
	Serum thyroxine (57.92–198.2 nmol/L)	–	–	–	–	–	131.11	–	–	–	–
	Free thyroxine (6.44–29.6 pmol/L)	–	18.28	13.26	–	–	11.49	–	–	–	–
	Serum triiodothyronine (1.08–3.38)	–	–	–	–	–	2.2	–	–	–	–
	Free triiodothyronine (2.73–8.6 pmol/L)	–	4.62	4.55	–	–	4.77		–	–	–

a *Oral hydrocortisone was started at 30 mg/m^2^ body surface area per day after the blood test*.

b *Blood was drawn before hydrocortisone intake*.

c *Blood was drawn 1 h after hydrocortisone intake*.

d *Follow-up testing results 3 weeks after stopping oral hydrocortisone therapy. ACTH, Adrenocorticotropic hormone; –, not available*.

**Figure 2:**
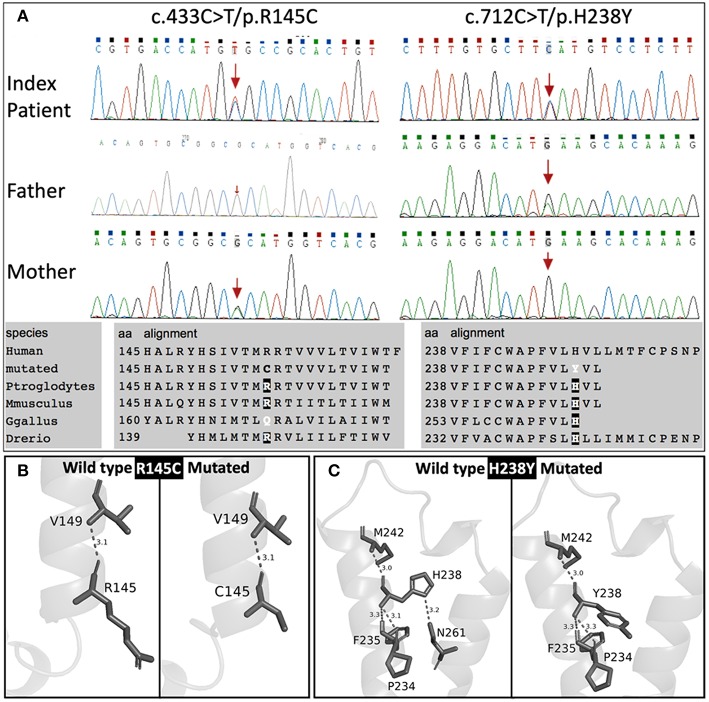
Sanger sequencing results with conservation status of amino acid residues **(A)**, and protein modulation **(B,C)**.

Genetic screening for abnormalities related to congenital adrenal hyperplasia (list of 44 genes are provided in Table 3), and multiplex ligation-dependent probe amplification (MLPA) analysis of the CYP21A2 gene were performed by a commercial genetic testing company (Customized target capture sequencing, http://www.mygenostics.com/ServiceTechnology.aspx?nid=263&pid=268). The result showed compound heterozygous variants in the melanocortin 2 receptor (*MC2R*) gene, but the result of the *CYP21A2* gene MLPA analysis was negative for hot-spot mutations and copy number variants (Supplementary Figure 1). We conducted protein modeling with SWISS-model (https://www.swissmodel.expasy.org) using the most similar structure (5jtb.1.A, Adenosine receptor A2a), and polar contacts of wild-type and mutated amino acid residues were compared with Pymol software (https://pymol.org/2/). The c.433C>T/p.R145C was reported in the dbSNP152 (http://www.ncbi.nlm.nih.gov/snp/rs139218324), and gnomAD (http://gnomad-old.broadinstitute.org/variant/18-13885085-G-A), but not in the 1000 Genome Database (http://www.1000genomes.org/) and Exome Variant Server (http://evs.gs.washington.edu/EVS/). The c.712C>T/p.H238Y variant was not reported in the dbSNP152, gnomAD, 1000 Genome Database, and Exome Variant Server. The c.433C>T/p.R145C variant of maternal origin caused the change of arginine (polar, basic) at the amino acid position of 145 to cysteine (non-polar, neutral). R145 is a relatively conserved amino acid residue, and five out of eight *in-silico* prediction tools (Table 2) predicted this variant as pathogenic. This is a known disease-causing variant (HGMD CM116421, rs139218324), and reported to be associated with FGD1 in an adopted Chinese girl (18). Protein modeling showed no effect of R145C residue change on polar contact with V149. The c.712C>T/p.H238Y variant of paternal origin caused the change of amino acid residue histidine (polar, basic) at the position of 238 to tyrosine (polar, neutral). H238 is a strictly conserved residue, and all eight *in-silico* prediction tools predicted this variant as pathogenic (Table 2). Protein modeling showed that the H238Y mutation changed polar contact of the amino acid residue in the position of 238 with adjacent residues, and polar contact with N261 in the transmembrane domain (TMD) 7 was lost. Confirmation with Sanger sequencing, conservation status of amino acid residue that have been affected, protein modeling results, and *in-silico* pathogenicity prediction results for both *MC2R* variants are provided in Figure 2 and Table 2.

**Table 2:** Carrier frequency and i*n-*silico pathogenicity prediction results of MC2R gene variants.

**Variant/Amino-acid (physical location)**	**Change**		**c.433C>T/p.R145C^a^ (chr18:13885085G>A)**		**c.712C>T/p.H238Y (chr18:13884806G>A)**
Carrier frequency (number of heterozygous/homozygous carriers)	1000G		0		0
	EXAC		0		0
	EXAC (East Asians)		0		0
	EXAC (South Asians)		0		0
	gnomAD		0.000007219 (2/0)		0
	gnomAD (East Asian)		0		0
	gnomAD (South Asian)		0		0
	gnomAD (African)		0.00004163 (1/0)		0
	gnomAD (European, non-finnish)		0.000007901 (1/0)		0
		**Prediction**	**Score**	**Prediction**	**Score**
*In silico* pathogenicity prediction (pathogenicity threshold)	Mutation taster (probability)	Disease causing	0.999	Disease causing	1.000
	SIFT (<0.05)	Damaging	0.014	Damaging	0.000
	Provean (<= -2.5)	Deleterious	−6.19	Deleterious	−5.77
	Polyphen-2 (>0.8)	Probably damaging	0.999	Probably damaging	1.000
	Revel (>0.5)	Benign	NA	Damaging	NA
	MutPred2 (>0.5)	Non-pathogenic	0.405	Pathogenic	0.842
	M-CAP (>0.025)	Likely benign	0.009	Possibly pathogenic	0.056
	CADD (>20)	Pathogenic	24.1	Pathogenic	26.8

a *Known disease mutation at this position (HGMD CM116421), rs139218324. URLs of in-silico prediction tools: Mutation taster (http://www.mutationtaster.org); SIFT&Provean (http://provean.jcvi.org/genome_submit_2.php); Polyphen-2 (http://genetics.bwh.harvard.edu/pph2/); Revel (Rare Exome Variant Ensemble Learner software); MutPred2 (http://mutpred.mutdb.org); M-CAP (http://bejerano.stanford.edu/MCAP/); CADD (https://cadd.gs.washington.edu/score)*.

**Table 3:** Diagnostic evaluation.

**Etiological assessment**	**Investigations performed (normal unless otherwise indicated)**
Infections	Complete blood count; Blood culture for bacteria and fungus; Serology for human Legionella, hepatitis B, hepatitis C, HIV, syphilis, Epstein-Barr virus (EBV), cytomegalovirus (CMV), herpes simplex viruses (HSV) I/II, toxoplasmosis, rubella virus, mycoplasma, chlamydia, tuberculosis, and respiratory syncytial virus; Complete blood count (slightly increased reticulocyte percentage) (Table 1). PCR for serum DNA detection for EBV, CMV, and HSV I/II; Elevated procalcitonin levels; Routine urine and fecal analyses.
Radiology and ultrasonography	Echocardiography (atrial septal defect, 3.7 mm), electroencephalography, electrocardiography, brain MRI, contrast MRI scan of the pituitary gland, chest X-ray (pneumonia), computed tomography scan of the brain. Bilateral hip ultrasound (possible hip dysplasia at when 5-days old, normal at 1.5 months); Abdominal ultrasound: slight hepatomegaly with decreased echogenicity (2–2.5 cm below the right costal margin, and 2.5 cm below the xiphoid process), slight splenomegaly (1.5–2.0 cm below the left costal margin); normal looking portal vein, pancreas, kidneys, and adrenal glands.
Immunology and coagulation profiles	Coomb's test at 5 days; Glucose-6-phosphate dehydrogenase (G6PD) activity at 5 days; INR and prothrombin time: slight elevation during newborn period, but normal afterwards); D-dimer (0.7–2.64 mg/L, normal range 0–0.3 mg/L); Activated partial thromboplastin time (46.1–51 s, normal range, 28.0–44.5 s); Fibrinogen (1.44–1.89 g/L, normal range, 2–4 g/L); Fibrinogen degradation products; Thrombin time.
Biochemical, metabolic and endocrine profiling	Liver function test (hyperbilirubinemia during newborn period, cholestasis), serum electrolytes, creatinine, uric acid, urea nitrogen, cholesterol, triglyceride (slightly elevated at 3.8 months) (Table 1) Serum creatine kinase (significantly elevated when 2 days old, normal afterwards), creatine kinase-MB, lactate dehydrogenase (slight elevation). Serum ceruloplasmin level at 1.5 months; Slightly elevated lactic acid and ammonia levels; Blood mass spectrometry at 1.6 months: elevated levels of oxalic acid (15.66 uM, normal range 0–0.1 uM), sebacic acid (24.38 uM, normal range 0.4–7 uM), 3-hydroxyglutaric acid (16.76, normal range 0–0.5 uM), palmitic acid (95.22 uM, normal range 0–13.8 uM) Low levels of 25-hydroxy vitamin D3 (10.41 ng/ml, normal range 15–35 ng/ml), and elevated levels of alphafetoprotein (7,575 ng/ml, normal range < 77.1 ng/ml) at 3.5 months. Serum amino acid and acyl-carnitine profiles at 3.5 months were normal except for slightly elevated threonine (126.35 uM, normal range 17–90 uM), and C0 (54.28, normal range 10–50 uM). Urine organic acid analysis (qualitative) at 3.5 months: significant elevation of citric acid, and slight elevation of 2-oxoglutaric acid and lactic acid. Glucose profiling (hypoglycemia); Low to normal levels of Insulin and C-peptide; Slightly elevated Serum lactate (Tables 1, 2); Hemoglobin A1c (2.2%, normal range 3.8–5.8%). Extremely low levels of serum cortisol, extremely high levels of serum ACTH; Lower level of 17-alpha hydroxyprogesterone, androstenediol, dehydroisoandrosterone; Higher levels of renin, aldosterone, angiotensin II, and dehydroepiandrosterone sulfate; Normal to higher levels of testosterone; Transient hypothyroidism (Table 2).
Genetic disorders	Genetic panel for screening for congenital adrenal hyperplasia including *NR0B1, PRKACA, DHCR7, GK2, PDE8B, LHX4, ARMC5, MC2R, GK, H6PD, CDKN1C, CYP11B1, ABCD1, SOX3, GNAS, MRAP, POMC, HSD11B1, MKS1, CYP21A2, NNT, TBX19, MEN1, MCM4, REN, NR5A1, AIRE, CYP17A1, NR3C1, PCSK1, TXNRD2, CYP11A1, RXRA, PRKAR1A, HESX1, HSD3B2, GLCC11, TP53, CYP11B2, POR, RXRB, PDE11A, PROP1*, and *STAR* gene (compound heterozygous variants in MC2R gene, Table 2, Figure 2) Multiplex ligation-dependent probe amplification (MLPA) analysis of CYP21A2 gene

Extensive etiologic evaluations from birth until the last follow-up (4.9 months) are provided in Table 3. After ruling out other causes of hypoglycemia, cholestasis, and adrenal deficiency, a diagnosis of FGD1 was made. Oral hydrocortisone was started at a dose of 30 mg/m^2^ body surface area (divided into three doses) at the age of 3.4 months. Cholestasis was resolved at 4.9 months, skin hyperpigmentation was improved, and no further episodes of hypoglycemia occurred. Morning serum cortisol levels 1 h after hydrocortisone intake was normal, while ACTH levels returned to near normal levels. However, parents decided to stop the medication at the age of 7.4-months, and serum cortisol/ACTH levels returned to extreme levels at the age of 8.1-months (Table 1).

## Discussion

ACTH unresponsiveness was first described by Shepard et al. (19) in 1959, melanocortin receptors were cloned in 1992 (20), and the first FGD1 caused by the *MC2R* gene mutation was reported by Clark et al. (2).

Hypothyroidism had been reported in an FGD1 patient with compound heterozygous L46fs/V49M mutation. The TSH level of 13.9 mIU/L at 3-months of age was normalized after a week of L-thyroxine therapy and remained normal when the medicine was stopped after 3 months (6). Our patient had hypothyroidism (TSH 10.61 mIU/L) during the neonatal period, but the repeated TSH levels without hormone replacement therapy at the age of 1.4 months was normal. Partial or complete deficiency of sex hormones such as 17-alpha hydroxyprogesterone, androstenediol, dehydroisoandrosterone, testosterone, progesterone, and dehydroepiandrosterone sulfate (DHES) had been reported in several FGD1 patients (4, 7, 9, 10, 13–15, 17). Besides low levels of androstenediol, DHES, and 17-alpha-hydroxyprogesterone, our patient also had lower levels of dehydroisoandrosterone and progesterone, as well as higher levels of testosterone. Although FGD1 patients may experience delayed development of pubic hair, other sexual characteristics did not seem to be affected. Slight abnormalities in renin or aldosterone levels have been reported (7, 8, 11, 13, 14), but angiotensin II levels have never been reported to be elevated in FGD1 patients. Renin, aldosterone, and angiotensin II levels were slightly elevated in our patient without any abnormalities in serum electrolytes, blood pressure, and kidney function. Patients with severe or homozygous truncating mutations in the *MC2R* gene, mild disturbances in renin–angiotensin–aldosterone axis may need temporary replacement of mineralocorticoid but did not cause long-term mineralocorticoid deficiency after stopping the intervention (11, 12).

A tall stature in the presence of normal growth hormone levels is one of the features of FGD1 (7, 8, 10, 14, 15, 17) accompanied by some dysmorphic features (such as hypertelorism, relative frontal prominence, epicanthic folds, large head circumference, and small tapering fingers) (7, 8). *In vitro* studies indicated that excess levels of ACTH increases chondrocyte precursors leading to chondrogenic phenotypes (21), and low levels of cortisol may fail to inhibit the synthesis of insulin-like growth factor-binding protein-5 (22). A single transverse palmar crease was associated with 107 genes and 172 disease entities (http://www.geneontology.org/formats/oboInOwl#id: HP:0007598), but not with the MC2R gene or FGD1. Since none of the transverse palmar associated genes were screened in this patient, we cannot rule out the possibility that variants in other genes may have caused this phenotype. Our patient had a normal body weight and length percentiles until hydrocortisone treatment, but both weight and length percentiles exceeded the 95th percentile 1.5 months post-treatment. This might be due to inadequate suppression and a prolonged effect of elevated ACTH. A tall stature is believed to return to normal after continuous treatment with hydrocortisone. However, growth parameters should be monitored in patients with FGD1, and growth hormone levels together with bone maturity should be evaluated when necessary. We also observed slight dysmorphic features, such as a prominent forehead, and hypertelorism, but no epicanthic folds were observed in our patient. Other endocrine abnormalities, and dysmorphic features in previously reported cases are summarized in Table 4.

**Table 4:** Other endocrine abnormalities and dysmorphic features in published cases.

**Cases**	**Amino acid change in MC2R protein**	**Gender, age (y, years; m, months; d, days)**	**Additional endocrine abnormalities**										**Other features**
			**Renin**	**Aldosterone**	**Angiotensin II**	**17-alpha hydroxy-progesterone**	**Andro-stenediol**	**Dehydro-isoandrosterone**	**Testosterone**	**Progesterone**	**Dehydro-epiandrost-erone Sulfate**	**Thyroid-stimulating hormone**	
Current report	R145C/H238Y	Female, 4.9 m	↑	N/↑	↑	↓/↓↓	N/↑	↓	N/↑	N/↑	↓	↑/N	Tall stature, prominent forehead, hypertelorism, and transverse palmar crease
Weber et al. (15)	S74I/R128C	Male, 8 y, 6 m	N	N	–	↓	N	–	N	–	–	–	Tall stature
	I44M/L192fs	Female, 2 y	N	N	–	↓	↓	↓	–	–	↓	–	Tall stature
	R146H/R146H	Female, 3 y, 4 m	–	–	–	–	–	–	–	–	↓	–	–
Tsigos et al. (16)	Y254C/Y254C	Female, 2 y, 3 m	N	N	–	–	–	–	–	–	–	–	Developmental delay
Naville et al. (13)	C251F/G217fs	Male, 2 y	N	↑	–	↓	–	–	–	–	–	–	–
	D107N/D107N	Male, 3 y	N	↑	–	–	–	–	–	–	–	–	–
Slavotinek et al. (8)	R146H/R146H	Female, 5 y, 9 m	↑	N	–	N	–	–	–	–	–	–	Tall stature, broad nasal bridge, small tapering fingers
Elias et al. (7)	T159K/T159K	Male, 8 d	N	↑	–	↓	–	–	–	–	–	–	Tall stature
	T159K/T159K	Male, 3 m	N	↑	–	–	–	–	–	–	–	–	
	T159K/D103N	Male, 2 y	N	↑	–	↓	–	–	–	–	–	–	Tall stature, advanced bone age, large head circumference, hypertelorism, epicanthic folds
	S74I/1052 delC	Male, 3 y	↑	N	–	↓	–	–	–	–	–	–	
	S74I/S74I	Male, 6 y	↓	–	–	–	–	–	–	–	–	–	
Selva et al. (17)	S74I/T159K	Female, 13 y, 8 m	N	–	–	N	–	↓	N	–	–	–	Short stature
Matsuura et al. (10)	C21Y/R146H	Female, 2 y	–	–	–	↓	–	–	–	–	–	–	Tall stature
Lin et al. (11)	S74I/S74I	Female,3 m	↑	↓	–	–	–	–	–	–	–	–	poor weight gain
	R146H/560delT	Male, 1 y, 7 m	↑	–	–	N	–	–	–	–	–	–	failure to thrive,
		Female, 1 y	N	↓	–	–	–	–	–	–	–	–	–
	579–581delTGT/579–581delTGT	Male, 2 m	↑	↓	–	–	–	–	–	–	–	–	–
Artigas et al. (14)	G217fs/A26S	Male, 2 y	↑	–	–	↓↓	–	–	↓	–	–	N	Tall stature
Mazur et al. (6)	L46fs/V49M	Male, 3 m	N	–	–	N	–	–	–	–	–	↑	Constipation, muscle weakness
Akin et al. (4)	L225R/L225R	Male, 7 d	N	N	–	↓↓	↓↓	–	–	–	↓↓	–	–

**Figure 3:**
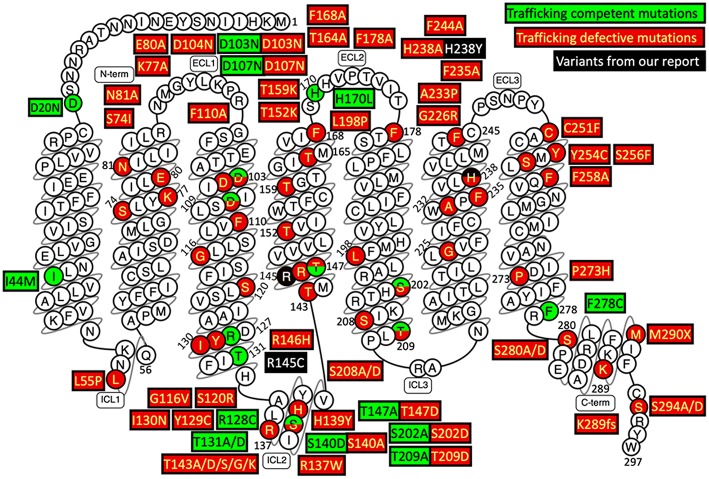
Naturally occurring and site-directed *MC2R* gene mutants their effect on protein function. This snake diagram of MC2R protein was obtained from G protein-coupled receptors database (GPCRdb, http://gpcrdb.org/family/001_002_014_002/).

N, normal; ↑, increased levels; ↑↑, significantly increased levels; ↓, decrease levels; ↓↓, significantly decrease levels; -, not available or not provided;

The underlying mechanism of the *MC2R* gene mutations causing FGD1 is ACTH resistance, either due to the trafficking failure of the receptor from the endoplasmic reticulum to the cell surface, or due to ineffective binding to ACTH (5). according to the last published review in 2018 (23), a total of 28 missense mutations, three non-sense mutations, and eight small insertion/deletions in the *MC2R* gene were reported in the literature. Most naturally occurring or site-directed mutants cause defective trafficking of the MC2R protein toward the cell membrane while others may lead to defective binding with ACTH (Figure 3). R145C found in our patient was previously reported in an adopted Chinese FGD1 child (18) and a known disease-causing variant in HGMD (CM116421), but no functional study was conducted to evaluate its effect in protein function. Located in the transmembrane domain 4 (TMD4), R145C is adjacent to R146H, which is also a naturally occurring disease causing mutant that causes decreased binding to ACTH and defective membrane trafficking. Site directed mutagenesis of another adjacent residue (T147D) resulted in a trafficking defect, but T147A did not affect protein trafficking toward the cell membrane (24). TMD4 plays a critical role in the activation of the rainbow trout melanocortin-2 receptor (25). R145C may have caused abnormal MC2R protein function by affecting receptor localization in the cell membrane, or activation of the receptor itself. H238Y (located in the TMD6) in our patient is a novel variant in a highly conserved residue not only among different species, but also among other melanocortin receptors. An *in vitro* study of site directed mutagenesis at the same amino acid residue (H238A) caused a 1.4-fold decrease in membrane trafficking of the MC2R protein from Golgi apparatus toward the cell membrane. H238 residue is also a component of the proposed ACTH binding site consisting of E80, D104, D107, F168, F178, F235, H238, and F258 (26). These findings suggest that the H238Y variant may affect cortisol production by affecting the MC2R localization and ACTH binding. Further functional studies are needed to prove variants found in our patient affect the MC2R protein function.

In conclusion, we reported on the first child with FGD1 from mainland China, carrying a novel *MC2R* variant, and reviewed previously reported cases with additional features.

## Informed Consent

Written informed consent was obtained from the parents of the participant for the publication of this case report.

## Ethics Statement

Ethical Committee of Children's Hospital of Fudan University approved the study.

## Author Contributions

J-SW designed the report and approved the final submission. KA and Z-DL collected data. KA analyzed relevant information and conduced protein modeling. Z-DL analyzed the next generation sequencing data. J-SW, KA, Z-DL, X-BX, Y-CL, and JZ, clinically managed the patient. Both KA and Z-DL wrote the manuscript, contributed equally for this manuscript, and will be first co-authors.

### Conflict of Interest Statement

The authors declare that the research was conducted in the absence of any commercial or financial relationships that could be construed as a potential conflict of interest.

## Supplementary Material

Supplementary Figure 1 CYP21A2 gene MLPA analysis was negative for hot-spot mutations and copy number variants.
