# Supplementary figures and images for: Novel Melanocortin 2 Receptor Variant in a Chinese Infant With Familial Glucocorticoid Deficiency Type 1, Case Report and Review of Literature
Source: Front Endocrinol (Lausanne). 2019 Jun 6;10:359. doi: 10.3389/fendo.2019.00359 (PMC6563654; doi:10.3389/fendo.2019.00359)

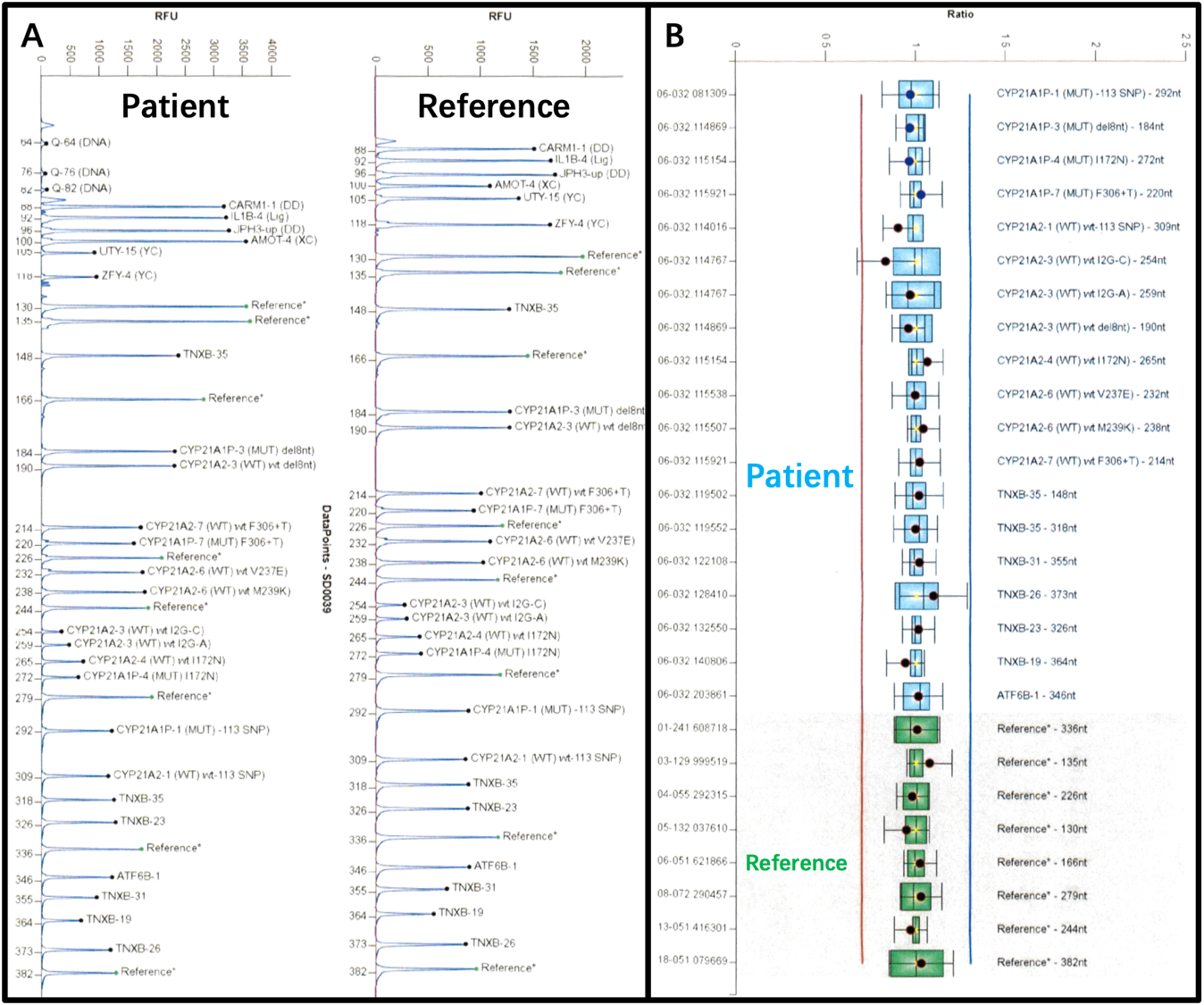

Supplement: Supplementary Figure 1 — CYP21A2 gene MLPA analysis was negative for hot-spot mutations and copy number variants. [file Image_1.PNG]
